# Assessment of the Probiotic Properties of *Yarrowia lipolytica* Isolated from Cold-Pressed Olive Oil

**DOI:** 10.3390/microorganisms12091905

**Published:** 2024-09-19

**Authors:** Pınar Keskin, Eda Kılıç Kanak, Suzan Öztürk Yılmaz

**Affiliations:** Department of Food Engineering, Sakarya University, Sakarya 54187, Türkiye

**Keywords:** *Y. lipolytica*, conventional olive oil, probiotic yeast

## Abstract

This research aimed to identify the probiotic features of *Yarrowia lipolytica* strains isolated from olive oils in Turkey. The in vitro survival capabilities of *Y. lipolytica* strains in gastric and pancreatic solutions were assessed. The hydrophobicity of *Y. lipolytica* strains was determined to be between 25.8% and 46.08% for xylene, 22.5% and 45.85% for chloroform, and 14.83% and 37.09% for ethyl acetate. In addition, auto-aggregation values were measured as 11.07–60.35%; 16.28–67.70% and 42.89–85.21% after 2, 4 and 24 h of incubation, respectively. The *Y. lipolytica* strains tested in this study demonstrated aggregation ability against the pathogens *Escherichia coli* ATCC 25922, *Salmonella* typhimurium ATCC 14028, *Staphylococcus aureus* ATCC 25923 and *Listeria monocytogenes* ATCC 7644. Antibiotic resistance and hemolytic activities were also checked to ensure the safety of the *Y. lipolytica.* Cholesterol removal by *Y. lipolytica* strains ranged from 12.30% to 47.42%, and their free radical scavenging activity varied between 2.85% and 39.10%. Out of 13 *Y. lipolytica* samples from 10 different olive oil sources, *Y. lipolytica* Y6, Y7, and Y11 exhibited the best strains with probiotic potential properties. This study discovered that *Y. lipolytica* with probiotic properties can be isolated in olive oil samples, a finding that has not been previously documented in the literature and may have potential industrial applications

## 1. Introduction

Probiotics are live microorganisms residing in the human gastrointestinal tract, recognized for their substantial and diverse beneficial effects on host health [1]. Most microorganisms accepted as probiotics are bacteria, particularly species of *Lactobacillus* and *Bifidobacterium* [2]. Some studies have indicated that the probiotic activities of bacteria can be unstable and that their resistance varies depending on environmental conditions. Examples of these variabilities include low survival rates at low pH, pathogenicity, antibiotic sensitivity, invasive nature, and toxin production. These disadvantages have pushed researchers to search for new probiotic microorganisms. The quest for more robust probiotic microorganisms has heightened interest in *Saccharomyces boulardii*, the first yeast demonstrated to have clinical effects and probiotic efficacy [3].

Yeasts offer several advantages as probiotics over bacteria, including higher survival rates in acidic gastric environments, resistance to bacterial antibiotics, antimicrobial activity against pathogenic microorganisms, and a lack of genetic material transfer [3,4]. Recent advances in yeast biology have increased interest in yeasts found in food. The growing use of yeasts is attributed to their numerous beneficial properties, with many strains being non-pathogenic to humans [5].

Lately, it has been determined that extra virgin olive oil contains yeasts. It is due to the transition of the carposphere from olive to olive oil during the extraction process of olives [6]. While some of these yeasts cannot survive for long in oily habitats, some reproduce selectively according to the product’s chemical composition, forming the characteristic microbiota of olive oil [7].

Studies on yeasts in extra virgin olive oil are limited, as their presence has only been detected recently [8]. Despite the multiple typical of many yeast strains isolated from foods, only *S. cerevisiae var. boulardii* a strain of *S. cerevisiae*, was accepted as probiotics and this has led the researchers to investigate the probiotic activities of other yeasts as well [7]. Because of its potential to produce lipase and protease, *Y. lipolytica* can be found in lipids- or protein-rich foods such as poultry, olive oil, cheese, and milk [9]. *S. cerevisiae* is widely used commercially as a probiotic, but yeast strains such as *Debaryomyces hansenii*, *Kluyveromyces lactis*, *Sterigmatomyces halophilus*, *Pichia pastoris* and *Y. lipolytica* also showed exceptional probiotic effects. The unique physiological properties of *Y. lipolytica* and the recognition of its generally safe status (GRAS) make this yeast important for biotechnological applications [10]. It has been noticed that *Y. lipolytica* positively influences the intestinal microbiota and reduces the number of pathogenic microorganisms in the intestine at a higher rate than *S. cerevisiae* [11]. It was also reported that *Y. lipolytica*, when combined with a probiotic, reduced the growth of *E. coli* and coliform bacteria in the intestinal contents, an effect was not observed with *S. cerevisiae* [12]. Reyes-Becerril et al. [13] observed that *Y. lipolytica* exhibits strong antioxidant and antimicrobial effects in their study of probiotic properties. Agarbati et al. [14] noticed that some of the *Y. lipolytica* isolated from cheeses in the Marche region of Italy showed high antioxidant properties.

Consumers usually ingest live yeast cells of olive and extra virgin olive oil. However, the role and behavior of oil-derived yeasts in the gastrointestinal tract remain poorly understood [15]. Given this knowledge gap, the purpose of this research is to investigate the probiotic and technological activities of yeasts isolated from cold-pressed olive oil products. Additionally, in many studies, probiotic yeasts are isolated from animal products, which prevents the use of probiotics in vegan products. In this study, these yeasts isolated from olive oil will not be problematic when using probiotics in vegan products.

## 2. Materials and Methods

### 2.1. Isolation of Yeast from Olive Oil

In this study, researchers collected 10 different olive oils produced via cold extraction methods, from regional grocery stores in Sakarya province of Türkiye. 10 mL of each sample was enriched in Sabouraud 2% Dextrose Broth (SDB) (Merck, Darmstadt, Germany) and incubated for 24 h at 27 °C under aerobic conditions. Seventy-four distinct colonies were randomly picked from Oxytetracyclin-Glucose-Yeast Extract Agar Base (OGYE) agar (Merck, Germany). Twenty-five colonies were identified as yeast under the microscope. After incubation, each isolated colony was stored at −60 °C in an SDB medium containing 20% glycerol. *S. boulardii* (Floratil^®^, Merck S.A., São Paulo, Brazil) microorganisms was used for the control.

### 2.2. Identification of Yeasts with MALDI-TOF MS

Yeasts were identified by MALDI-TOF MS (Matrix Assisted Laser Desorption/Ionization Time-of-Flight Mass Spectrometry, Bruker, Karlsruhe, Germany). Yeast isolates were spread on a 96-well plate, 1 µL (70% formic acid) was added and left to dry. Then, 1 µL of the matrix was dropped onto the sample and dried again. Further analyses were determined for 13 strains identified as *Y. lipolytica.*

### 2.3. Molecular Identification of Yeast

Total genomic DNA of yeasts was detected using the EZ1 DNA Tissue Kit (Qiagen, Hilden, Germany). The D1/D2 region of the large subunit of yeast 26S rDNA was amplified with the universal primers, 5′-GCA TAT CAA TAA GCG GAG GAA AAG-3′ and 5′-GGTCCGTGTTTCAAGACGG-3′ for forward and reverse, as described by Kurtzman and Robnett [16].

### 2.4. Characterization of Probiotic Properties

#### 2.4.1. The Ability of Yeast to Survive under In Vitro Gastric Digestion

The survival abilities of yeasts in gastric fluid environments were assessed [17]. A synthetic gastric fluid was prepared using a buffer solution at pH 2.0 (adjusted with 1 M HCl), containing [(NaCl (2.05 g/L), KH_2_PO_4_ (0.60 g/L), CaCl_2_ (0.11 g/L), and KCl (0.37 g/L)]. After preparing the mixture, pepsin (0.0133 g/L) and lysozyme (0.01 g/L) were added to obtain the gastric solution. Yeast cultures were grown on PDA (Merck, Germany) (25 °C for 24 h), then developed in TSB yeast broth, centrifuged (5000× *g*, 10 min, 4 °C), and the pellets were washed twice with sterile 0.9% NaCl solution (*w*/*v*) and resuspended in sterile 0.9% NaCl solution (*w*/*v*). Subsequently, cells were resuspended in the gastric solution, adjusted to an optical density (OD) of 0.5 (λ = 600 nm) with 0.9% NaCl (lof CFU before), and incubated at 37 °C for 2.5 h with orbital shaking at 200 rpm to simulate peristaltic movements (log CFU after). Cell viability was evaluated at the beginning and the end of each digestion simulation by incubating on PDA culture medium at 25 °C for 2 days. The survival percentages of the samples in the gastric liquid environment were calculated.

#### 2.4.2. The Ability of Yeast to Survive under In Vitro Pancreatic Juice

The simulated pancreatic juice was applied with minor modifications [17]. The juice was prepared at pH 8.0 using a buffer solution prepared with bile salts (3.0 g L^−1^), pancreatin (0.1 g L^−1^), Na_2_HPO_4_7H_2_O (50.81 g L^−1^) and NaCl (8.5 g L^−1^). Cells collected in the gastric digestion analysis were rewashed for this analysis and added to the prepared pancreatic juice solution (lof CFU before). Incubated at 200 rpm, 37 °C for 3.5 h (log CFU after), samples were counted using OGYE agar at the beginning and end of each gastric digestion.

#### 2.4.3. Cell Surface Hydrophobicity

The adhesion of yeast isolates to hydrocarbons was applied with some modifications [18] The isolates were activated in Tryptic Soy Broth (TSB) (Merck, Germany) by incubation at 37 °C for 48 h. The yeasts were centrifuged at 5000× *g* for 10 min at 4 °C, and the pellets were washed twice with phosphate-buffered saline (PBS) and re-suspended in 0.1 M KNO_3_ (pH 6.2) buffer to give a final OD_600_ nm of 0.5.

2 mL of the yeast suspension was combined with 0.5 mL solvent non-polar neutral solvent (xylene), monopolar acidic solvent (chloroform) and basic monopolar solvent (ethyl acetate) and incubated at 37 °C for 30 min. After pre-incubation at room temperature, the two phases were mixed with a vortex for 2 min and left at room temperature for 4 h. After incubation, the aqueous phase was separated and the absorbance was detected at 600 nm.

$$\mathrm{Hydrophobicity}\left(\%\right)=1-\frac{A}{A0}\times 100$$

#### 2.4.4. Auto-Aggregation Capacity

The auto-aggregation capacity was determined with minor modifications [19]. Yeast isolates were incubated for 48 h at 37 °C and then centrifuged at 5000× *g* for 10 min at 4 °C. The yeast pellet was washed twice with PBS and re-suspended to give a final OD_600_ nm of 0.5. The resulting suspensions were mixed for 15 s and incubated at 37 °C. The supernatant’s optical density (OD) was measured at 600 nm at 0, 2, 4 and 24 h.

$$\mathrm{Autoaggregation}\left(\%\right)=1-\frac{\mathrm{At}}{A0}\times 100$$

#### 2.4.5. Co-Aggregation Capacity

The co-aggregation capacity of yeast isolates with *Escherichia coli* ATCC 25922 (clinical isolate from the American Type Culture Collection), *Listeria monocytogenes* ATCC 7644, *Staphylococcus aureus* ATCC 25923, *Salmonella* typhimurium ATCC 14028 strains was determined [20]. Thirteen yeast strain suspensions were arranged for the auto-aggregation experiment. Equal amounts of yeast culture (2 mL) and pathogen suspensions (2 mL) were mixed in the same glass test tube with a vortex for 10–15 s. Control tubes included a 2 mL suspension of individual bacterial strains. They were incubated at 24 °C for 24 h. After incubation, 0.1 mL was taken from the upper part of the culture mixtures, and the supernatant’s optical density (OD) was evaluated at OD600 nm. The co-aggregation activities of yeast samples were evaluated using the following equation.

$$\mathrm{Co}\text{-}\mathrm{aggregation}\;\left(\%\right)=\frac{\frac{\mathrm{Ax}+\mathrm{Ay}}{2}-A\left(x+y\right)\;}{\frac{\mathrm{Ax}+\mathrm{Ay}}{2}}\times 100$$

#### 2.4.6. Antimicrobial Activity

Determination of the antimicrobial activity of yeasts against pathogens was assessed in a slightly different mode than made by Syal and Vohra [21]. *E. coli* ATCC 25922, *St. aureus* ATCC 25923, *L. monocytogenes* ATCC 7644, *S*. *typhimurium* ATCC 14028 strains were grown in a Trypticase soy broth (Merck, Germany) at 37 °C for 24–48 h. The strains were incubated at 25 °C for 24 h in a Mueller–Hinton Agar (Merck, Germany). The cell-free supernatant was centrifuged at 5000× *g* for 10 min. Pathogen cultures (100 µL, 10^7^ CFU mL^−1^) were incubated in a TSA plate. 10 µL of a cell-free supernatant was poured onto the plates. The inhibition zone was evaluated after 24 h at 37 °C.

#### 2.4.7. Hemolytic Activity

Thirteen *Y. lipolytica* strains were measured for 48 h at 37 °C for cultures incubated on media containing 7% (*v*/*v*) sheep blood onto blood agar plates (Salubris, ABD, Shenzhen, China) [22].

#### 2.4.8. Antibiotic Susceptibility

Thirteen *Y. lipolytica* strains were determined using the Kirby Bauer disk diffusion method. Each strain was grown in TSB yeast broth at 25 °C for 24 h. 100 μL of the culture (10^7^ CFU mL^−1^) was spread onto Mueller–Hinton agar plates (Merck, Germany) and antimicrobial agent discs (Oxoid, Basingstoke, UK) were placed on the surface. The zone of inhibition was determined after incubating at 37 °C for 24 h. Four antimicrobial susceptibility test disks were used: erythromycin (15 µg), gentamicin (120 μg), tetracycline (30 μg) and vancomycin (30 μg), as determined by NCCLS protocol [23].

#### 2.4.9. Cholesterol Removal

The removal of cholesterol was performed [24]. The percentage of cholesterol removal was measured according to the following equation:

$$\mathrm{Cholesterol}\;\mathrm{removal}\;\left(\%\right)=1-\frac{A}{A0}\times 100$$

#### 2.4.10. The Antioxidant Activity Potential

The antioxidant activity of the yeasts was determined using the DPPH method [25]. Yeasts incubated at 25 °C for 24 h in TSB yeast were first centrifuged (5000× *g*, 10 min, 4 °C). The resulting pellets were then washed twice in PBS buffer (pH 7.4, OD600 nm 0.400 ± 0.05) and resuspended. Yeasts (2 mL) were mixed with 2 mL freshly prepared DPPH (0.2 mM in methanol), incubated in the dark for 30 min, and then centrifuged (5000× *g*, 10 min). The absorbance of the resulting supernatant was measured at 517 nm. Yeast-free samples were used as controls. The DPPH radical scavenging activity was determined using the following formula:

$$\%\mathrm{DPPH}\;\mathrm{radical}\;\mathrm{scavenging}\;\mathrm{ability}=1-\frac{\mathrm{OD}\;\mathrm{sample}}{\mathrm{OD}\;\mathrm{control}}\times 100$$

The Trolox equivalent antioxidant capacity of each yeast isolated was determined using a solution of 6-hydroxy-2,5,7,8-tetramethylchroman-2-carboxylic acid (Trolox) in methanol (1 mM).

### 2.5. Statistical Analysis

All the experiments were determined in three independent replicates and data were expressed as the means ± standard deviations. Data were analyzed using Student’s *t*-test and one-way analysis of variance ANOVA in the Statistical Package for Social Sciences (Ver. 19.0 SPSS, Chicago, IL, USA) at a significance level of *p* < 0.05.

## 3. Results

### 3.1. Identification of Y. lipolytica Isolates

Twenty-five yeasts were isolated from olive oils and identified as genotypes with MALDI-TOF MS Biotyper. Twenty-one were identified as *Y. lipolytica* and four were identified as *Wickerhamomyces anomalous*. Thirteen *Y. lipolytica* isolates were confirmed by 26S rRNA gene sequencing (Table 1).

### 3.2. The Ability of Yeast to Survive under In Vitro Gastric Digestion Pancreatic Juice

The growth ability of 13 strains under gastric digestion and pancreatic juice conditions in vitro was determined. Table 2 was also used to show which isolates were obtained from specific olive oils. conditions in vitro was determined. Over 60% of all *Y. lipolytica* yeasts survived in gastric digestion and pancreatic juice conditions (Table 2). Additionally, two strains of *Y. lipolytica Y5* and *Y. lipolytica Y9* were found to show higher survival rates under gastric digestion and pancreatic juice conditions than *S. boulardii* used as a control (*p* < 0.05). *Y. lipolytica* Y2, Y5, Y7, Y9, Y10, Y12 and Y13 strains survived better than *S. boulardii* in pancreatic juice (*p* < 0.05).

### 3.3. Cell Surface Hydrophobicity

This research evaluated the hydrophobicity of the *Y. lipolytica* strains (Table 2). The hydrophobicities of the *Y. lipolytica* was between 25.8 and 46.08%, 22.50 and 45.85% and 14.83 and 37.09% for xylene chloroform and ethyl acetate, respectively. Therefore, our results demonstrated that 13 *Y. lipolytica* isolates exhibited high colonization activity in the intestine. This research exhibited the highest cell hydrophobicity rate for strains *Y. lipolytica* Y6 and *Y. lipolytica* Y10. Compared to *S. boulardii* as a control, hydrophobicity in xylene and chloroform was lower in all yeasts (*p* < 0.05), while ethyl acetate was at higher rates (*p* < 0.05).

### 3.4. Co-Aggregation Capacity

All *Y. lipolytica* strains in this study exhibited the capacity to co-aggregation with the *E. coli* ATCC 25922, *L. monocytogenes* ATCC 7644, *S. aureus* ATCC 25923 and *S.* typhimurium ATCC 14028 (NY, US). The values of the co-aggregation of *Y. lipolytica* strains are presented in Table 2. Compared to *S. boulardii*, co-aggregation ability with *E. coli* was found to be higher in all yeasts except *Y. lipolytica* Y3, Y13 (*p* < 0.05). The co-aggregation ability with *L.monocytogenes* was found to be higher in other yeasts than *S. boulardi* except *Y. lipolytica* Y2, Y3 and Y9 (*p* < 0.05). The co-aggregation ability with *S. aureus* was found to be higher in other yeasts than *S. boulardii*, except *Y. lipolytica* Y9 and Y10 (*p* < 0.05). When the co-aggregation ability against *S*. typhimurium was examined, *S. boulardii* showed higher co-aggregation than all other yeasts (*p* < 0.05).

### 3.5. Auto-Aggregation Capacity

**Figure 1:**
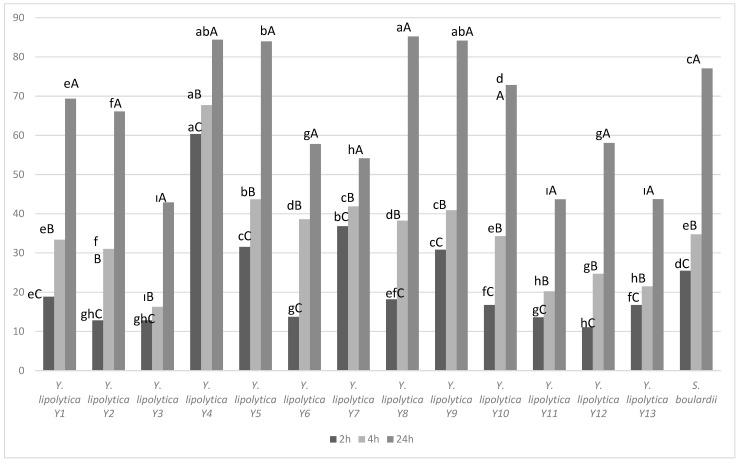
Auto-aggregation percentage after 2 h, 4 h and 24 h of *Y. lipolytica* strains and *S. boulardii.* Lowercase letters indicate the statistical difference between yeasts at the same time, uppercase letters indicate the difference between yeasts at different times.

All the strains of *Y. lipolytica* showed high auto-aggregation abilities. Auto-aggregation rates of the *Y. lipolytica* strains were in the range of 11.07–60.35%; 16.28–67.70% and 42.89–85.21% after 2 h, 4 h and 24 h incubation, respectively (Figure 1). This research exhibited the highest cell auto-aggregation rate for strain *Y. lipolytica* Y4. Compared to *S. boulardii*, higher auto-aggregation was detected in *Y. lipolytica* Y4, Y5, Y8 and Y9 strains at the end of 24 h (*p* < 0.05).

### 3.6. Antimicrobial Activity

The antimicrobial activity results are given in Table 3. *Y. lipolytica* strains were found have good antimicrobial activity against *E. coli* ATCC 25922, *L. monocytogenes* ATCC 7644, *St. aureus* ATCC 25923 and *S. typhimurium* ATCC 14028. The inhibition zones range from 0 to 19 mm. *Y. lipolytica* Y11 exhibited the strongest antimicrobial activity (18.75 mm) against *S. aureus* ATCC 25923, while *Y. lipolytica* Y11 also showed strong antimicrobial activity (19 mm) against *E. coli* ATCC 25922 (Table 3). Compared to *S. boulardii*, yeasts showed less antimicrobial effect against *S. aureus*, *E. coli* and *S. typhimurium* (*p* < 0.05), but showed higher antimicrobial effect against *L. monocytogenes* (*p* < 0.05).

### 3.7. Hemolytic Activity and Antibiotic Susceptibility

The *Y. lipolytica* exhibited no β-hemolytic activity (Table 4). Pathogens can use hemoglobin as a source of ferrous. They were evaluated for signs of β-hemolysis (light-colored zones around the colony), α-hemolysis (green zone around the colony), and γ-hemolysis (no zone).

Four antimicrobial agents were used to detect antibiotic resistance of *Y. lipolytica* strains. All strains were found to be susceptible to most of the antibiotics tested (Table 4). It was evaluated according to the breakpoints determined by NCCLS (National Committee for Clinical Laboratory Standards, 2000). All tested strains were not found to be resistant to erythromycin (15 µg), gentamicin (120 μg), tetracycline (30 μg) and vancomycin (30 μg) (Table 4).

### 3.8. Cholesterol Removal

In our study, *Y. lipolytica* isolates especially isolates *Y. lipolytica* Y6 (47.42 ± 0.09%) and *Y. lipolytica* Y3 (47.05 ± 0.26) were found to have the desired cholesterol-lowering ability. Cholesterol removal ranged from 47.42 ± 0.09% to 12.30 ± 0.11% (Table 5). Compared to *S. boulardii*, *Y. lipolytica* yeasts were found to have less cholesterol-lowering ability (*p* < 0.05).

### 3.9. Antioxidant Activity Potential

*Y. lipolytica* yeasts showed free radical scavenging activity in the range of 2.85–39.10%. *Y. lipolytica* Y3 (39.10 ± 0.84%) showed the highest antioxidant capacity (Table 5). Compared to *S. boulardii*, *Y. lipolytica* yeasts showed less antioxidant activity (*p* < 0.05).

## 4. Discussion

*Y. lipolytica* strains derived from cold-pressed olive oil performed well in vitro against gastric juice and intestinal juice. It was determined that the yeast did not have hemolytic activity. They were determined to be safe according to their antibiotic susceptibility profiles against erythromycin (15 µg), gentamicin (120 μg), tetracycline (30 μg), vancomycin (30 μg). Moreover, these strains performed well antimicrobial activity against various pathogens. They have also been found to have antioxidant activity and cholesterol removal abilities. In this research, 13 *Y. lipolytica* strains displayed promising probiotic potential, among which *Y. lipolytica* Y6, *Y. lipolytica* Y7, and *Y. lipolytica* Y11 exhibited the best probiotic features. For these strains, the percentage of co-aggregation, auto-aggregation and survival in gastric and intestinal juice is higher than 30% and they have a hydrophobicity rate greater than 30%. They are sensitive to antibiotics. They also have an antimicrobial effect.

Probiotic strains must pass through the stomach and small intestine. The pH in the stomach ranges from 2.5 to 3.5. Yeasts exhibit a remarkable ability to survive across a broad pH spectrum, with certain rare species demonstrating resilience even in highly acidic environments, enduring conditions as extreme as pH 1.5 [26].

Bonatsou et al. [17] determined that the overall survival rate of 42 of 49 yeast strains was 50% and above during simulated gastric and pancreatic digestions of yeasts. In our study, all *Y. lipolytica* yeasts survived in over 60% of gastric and pancreatic juice. Tolerance to in vitro gastric and pancreatic digests is essential for potential probiotic candidates. Meanwhile, some studies have investigated the probiotic potential of yeasts isolated from olives [17,27]. Additionally, two strains of *Y. lipolytica Y5* and *Y. lipolytica Y9* showed higher survival rates under gastric digestion and pancreatic juice conditions than *S. boulardii* used as a control (*p* < 0.05). *Y. lipolytica* Y2, Y5, Y7, Y9, Y10, Y12 and Y13 strains survived better than *S. boulardii* in pancreatic juice (*p* < 0.05) (Table 2).

Bonatsou et al. [17] determined that most of the yeasts found in the natural fermentation of black olives in Kalamata have a 75% and above hydrophobic activity. Yeast isolates obtained from Italian extra virgin olive oil showed the highest hydrophobicity of 55.50% [28]. The hydrophobic activities of yeast isolates obtained from pineapple peel and pulp were examined and the highest hydrophobicity value was determined as 99.66% [15]. The hydrophobic activities of yeast isolated from Nigerian grain-based traditional fermented food products were determined similar to our results between 33 and 42% [29]. In this study, compared to *S. boulardii* as a control, hydrophobicity in xylene and chloroform was lower in all yeasts (*p* < 0.05), while ethyl acetate was at higher rates (*p* < 0.05) (Table 2).

The capacity to adhere to hydrocarbons is involved in cell surface hydrophobicity and determines its capacity to adhere to intestinal epithelial tissue. Hydrophobicity is insufficient for adhesion to the epithelium alone but should be evaluated together with the aggregation feature. The hydrophobicity ability indicates the binding of probiotics to epithelial cells. As the hydrophobicity increases, the health benefits also increase [30]. Thanks to probiotics that adhere to the intestinal surface, pathogens cannot colonize the intestinal surface [31]. Probiotics that adhere to the intestinal surface take up nutrients. In addition, the organic acids and antimicrobial compounds they produce prevent the growth of pathogens [32].

Ogunremi et al. [29] observed that the auto-aggregation rate of *Pichia kluyveri*, *Isaatchenkia orientalis* and *Pichia kudriavzevi* yeasts, which they obtained from Nigerian traditional fermented food products, increased as the incubation time increased. This result is similar to our results. When auto-aggregation activities were examined in yeast isolates obtained from pineapple, the auto-aggregation capacity of all samples was below 16% after 2 h of incubation. This rate increased to 96% after 24 h of incubation [15]. Bonatsou et al. [17] determined that the auto-aggregation ability of the yeasts in the natural fermentation of black olives in Kalamata is between 72 and 91%. Compared to *S. boulardii*, higher auto-aggregation was detected in *Y. lipolytica* Y4, Y5, Y8 and Y9 strains at the end of 24 h (*p* < 0.05) (Figure 1).

In this study, it was determined that the co-aggregation capacity was dependent on the incubation time and strain. The co-aggregation activities of yeast strains isolated from traditional Nigerian cereal-based fermented food products were found to be between 57 and 71% [29]. The values of the co-aggregation of *Y. lipolytica* strains are given in Table 2. Compared to *S. boulardii*, co-aggregation ability with *E. coli* was found to be higher in all yeasts except *Y. lipolytica* Y3, Y13 (*p* < 0.05). The co-aggregation ability with *L. monocytogenes* was found to be higher in other yeasts than *S. boulardi* except *Y. lipolytica* Y2, Y3 and Y9 (*p* < 0.05). The co-aggregation ability with *S. aureus* was found to be higher in other yeasts than *S.boulardii*, except *Y. lipolytica* Y9 and Y10 (*p* < 0.05). When the co-aggregation ability against *S*. *typhimurium* was examined, *S. boulardii* showed higher co-aggregation than all other yeasts (*p* < 0.05) (Table 2).

The antimicrobial properties of yeast contribute to extending the shelf life of foods. Additionally, it is essential that the probiotic strain has a competitive advantage and prevents colonization of the intestine by pathogens. Silva et al. [27] determined that yeasts isolated from Portuguese olives exhibited antimicrobial efficacy against *E. coli*, *L. monocytogenes*, *S. enteritidis* and *S. aureus* pathogens). Compared to *S. boulardii*, yeasts showed less antimicrobial effect against *S. aureus*, *E. coli* and *S. typhimurium* (*p* < 0.05), but showed higher antimicrobial effect against *L. monocytogenes* (*p* < 0.05) (Table 3).

In order to determine the antibiotic resistance of yeasts obtained from pineapple peel and pulp, streptomycin, tetracycline, erythromycin, chloramphenicol, penicillin and ampicillin antibiotics were used and all yeasts showed resistance to antibiotics [15]. Perricone et al. [33] found that yeast isolates isolated from sourdough showed resistance to erythromycin, gentamicin, streptomycin, chloramphenicol and tetracycline antibiotics. In this study, *S. boulardii* were not found to be resistant to erythromycin (15 µg), gentamicin (120 µg), tetracycline (30 µg) and vancomycin (30 µg), similar to *Y. lipolytica* strains (*p* > 0.05) (Table 4).

Some medications are used to treat hypercholesteremia. These drugs may have some side effects in the gastrointestinal tract [34]. Therefore, taking advantage of probiotics is considered a good alternative. *S. boulardii* was found to have a higher cholesterol-lowering ability than *Y. lipolytica* strains (*p* < 0.05) (Table 5).

Probiotic microorganisms may protect against stomach ulcers, obesity, cardiovascular and chronic diseases thanks to their antioxidant effects [35]. We measured the antioxidant potential of *Y. lipolytica* strains in this study by DPPH radical scavenging. *S. boulardii* exhibited higher DPPH radical scavenging activity than *Y. lipolytica* strains (*p* < 0.05) (Table 5).

No other study has been found in the literature examining the probiotic potential of *Y. lipolytica* yeasts obtained from olive oil. Therefore, this study adds a new yeast with probiotic potential to the literature. Additionally, in many studies, probiotic yeasts are isolated from animal products, which prevents the use of probiotics in vegan products. In this study, these yeasts isolated from olive oil will not be problematic when using in vegan products. The strains with probiotic potential *Y. lipolytica* yeasts obtained from this study can be used for functional vegan food development in future studies. Valuable insights into the functional features of probiotics in cold-pressed olive oil have been obtained. Consequently, it can be concluded that conventional olive oil may also serve as a medium for screening and isolating probiotic and starter culture strains.

## Figures and Tables

**Table 1:** Identified matches of *Y. lipolytica* isolates by D1/D2 region sequencing analysis from GenBank database and MALDI-TOF MS Biotyper.

Matches to D1/D2 Sequences from GenBank Database	Accession No	Identity (%) with GenBank Database	MALDI-TOF MS	MALDI-TOF MS Score
*Y. lipolytica* Y1	MK358172	99	*Y. lipolytica*	1.959
*Y. lipolytica* Y2	MK358177	98	*Y. lipolytica*	1.854
*Y. lipolytica* Y3	MK358181	97	*Y. lipolytica*	1.954
*Y. lipolytica* Y4	MZ314862	97	*Y. lipolytica*	1.958
*Y. lipolytica* Y5	MH545931	95	*Y. lipolytica*	1.945
*Y. lipolytica* Y6	MN075241	95	*Y. lipolytica*	2.054
*Y. lipolytica* Y7	MK394170	95	*Y. lipolytica*	2.214
*Y. lipolytica* Y8	MH794379	98	*Y. lipolytica*	2.156
*Y. lipolytica* Y9	MH481651	95	*Y. lipolytica*	1.975
*Y. lipolytica* Y10	MH752200	99	*Y. lipolytica*	1.965
*Y. lipolytica* Y11	MH752198	95	*Y. lipolytica*	2.214
*Y. lipolytica* Y12	MH656399	97	*Y. lipolytica*	2.015
*Y. lipolytica* Y13	LC413272	96	*Y. lipolytica*	1.985

**Table 2:** The percentage of survival in an in vitro gastric solution, hydrophobicity and co-aggregation of *Y. lipolytica* strains from cold-pressed olive oil and *S. boulardii*.

		In Vitro Gastric Solution	In Vitro Pancreatic Juice	Hydrophobicity (%)			Co-Aggregation Percentage (%)			
Olive Oil Province	Strains			Xylene	Chloroform	Ethyl Acetate	*E. coli*	*L. mono*	*S. typhimurium*	*S. aureus*
Balıkesir	*Y. lipolytica* Y1	74.90 ± 0.02	84.60 ± 0.11	32.88 ± 3.35	22.76 ± 3.12	17.75 ± 2.88 *	72.80 ± 0.05 *	31.00 ± 0.13 *	15.70 ± 0.43	58.00 ± 0.02 *
Balıkesir	*Y. lipolytica* Y2	88.09 ± 0.12	90.90 ± 0.15 *	28.65 ± 2.49	22.50 ± 2.78	14.83 ± 2.26 *	57.50 ± 0.01 *	6.50 ± 0.16	12.20 ± 0.12	56.82 ± 0.05 *
Manisa	*Y. lipolytica* Y3	95.79 ± 0.47	75.80 ± 0.30	30.98 ± 2.03	33.85 ± 2.29	23.47 ± 1.80 *	46.20 ± 0.01	6.81 ± 0.13	17.19 ± 0.42	67.09 ± 0.01 *
Gemlik	*Y. lipolytica* Y4	87.51 ± 0.04	86.71 ± 0.07	29.55 ± 2.98	32.68 ± 2.93	28.96 ± 2.95 *	69.00 ± 0.23 *	51.04 ± 0.14 *	12.40 ± 0.20	60.00 ± 0.01 *
Gemlik	*Y. lipolytica* Y5	97.43 ± 0.15 *	98.62 ± 0.07 *	26.71 ± 0.78	31.90 ± 0.72	26.25 ± 0.65 *	49.60 ± 0.01 *	51.00 ± 0.16 *	13.70 ± 0.03	74.97 ± 5.29 *
Datça	*Y. lipolytica* Y6	88.99 ± 0.11	60.49 ± 0.01	46.08 ± 0.08	45.85 ± 0.04	32.51 ± 0.08 *	63.60 ± 3.07 *	14.40 ± 0.15 *	11.00 ± 0.48	62.17 ± 0.08 *
Marmaris	*Y. lipolytica* Y7	79.93 ± 0.06	93.65 ± 0.12 *	41.77 ± 0.40	31.90 ± 0.36	31.34 ± 0.33 *	63.70 ± 0.15 *	8.11 ± 0.13 *	12.60 ± 0.45	68.36 ± 0.00 *
Marmaris	*Y. lipolytica* Y8	88.68 ± 0.14	74.09 ± 0.03	26.43 ± 0.22	26.12 ± 0.28	37.09 ± 0.27 *	52.90 ± 0.08 *	11.90 ± 0.16 *	11.80 ± 0.06	32.00 ± 0.05
Hatay	*Y. lipolytica* Y9	97.14 ± 0.28 *	98.52 ± 0.07 *	26.31 ± 0.08	36.49 ± 0.13	25.79 ± 0.12 *	64.80 ± 0.26 *	4.96 ± 0.15	15.00 ± 0.04	25.33 ± 0.00
Edremit	*Y. lipolytica* Y10	73.81 ± 0.09	95.08 ± 0.17 *	25.08 ± 0.07	44.82 ± 0.02	34.32 ± 0.01 *	73.70 ± 0.06 *	16.90 ± 0.12 *	16.00 ± 0.43	56.80 ± 0.06 *
İzmir	*Y. lipolytica* Y11	74.17 ± 0.00	74.19 ± 0.13	34.10 ± 0.09	33.85 ± 0.14	34.29 ± 0.13 *	64.40 ± 0.32 *	10.90 ± 0.12 *	12.90 ± 0.48	71.10 ± 0.06 *
Sakarya	*Y. lipolytica* Y12	86.58 ± 0.25	94.25 ± 0.29 *	31.34 ± 0.25	28.06 ± 0.30	25.72 ± 0.26 *	67.50 ± 0.28 *	8.28 ± 0.14 *	14.40 ± 0.02	48.51 ± 0.02 *
Germencik	*Y. lipolytica* Y13	75.09 ± 0.34	96.79 ± 0.09 *	34.84 ± 0.05	31.55 ± 0.10	25.97 ± 0.09 *	47.00 ± 0.00	8.14 ± 0.11 *	10.00 ± 0.05	57.62 ± 0.07 *
	*S. boulardii*	95.45 ± 0.48	88.75 ± 0.17	47.67 ± 0.74	80.74 ± 1.58	10.46 ± 0.86	47.10 ± 0.24	7.62 ± 0.16	20.10 ± 0.54	41.26 ± 0.06

For each sample, the mean of three values is presented ± SD. *: indicates statistically significant difference between control and all experimental groups as evaluated by Student’s *t*-test (*p* < 0.05), *S. boulardii*: control.

**Table 3:** Antimicrobial activities of *Y. lipolytica* strains obtained from cold-pressed olive oil and *S. boulardii*.

Strains	*S. aureus* ATCC 25923	*L. monocytogenes* ATCC 7644	*E. coli* ATCC 25922	*S. typhimurium* ATCC 14028
*Y. lipolytica* Y1	17.50 ± 2.50	11.00 ± 1.00 *	13.75 ± 0.70	12.00 ± 0.00
*Y. lipolytica* Y2	16.00 ± 2.00	9.00 ± 0.00 *	14.00 ± 2.00	7.00 ± 3.00
*Y. lipolytica* Y3	6.00 ± 2.00	11.00 ± 1.00 *	10.75 ± 0.20	7.50 ± 2.50
*Y. lipolytica* Y4	14.25 ± 1.20	6.50 ± 2.50 *	11.25 ± 2.20	5.50 ± 2.50
*Y. lipolytica* Y5	7.00 ± 1.00	13.75 ± 1.70 *	7.25 ± 2.20	11.50 ± 0.50
*Y. lipolytica* Y6	00.00 ± 0.00	11.00 ± 0.00 *	13.00 ± 2.00	5.50 ± 0.50
*Y. lipolytica* Y7	00.00 ± 0.00	6.00 ± 2.00 *	4.50 ± 2.50	10.50 ± 1.50
*Y. lipolytica* Y8	8.50 ± 2.50	2.25 ± 0.70 *	4.25 ± 1.20	4.50 ± 2.50
*Y. lipolytica* Y9	14.00 ± 2.00	00.00 ± 0.00	8.25 ± 2.20	6.00 ± 2.00
*Y. lipolytica* Y10	7.50 ± 2.50	7.00 ± 2.00 *	10.25 ± 1.70	5.50 ± 2.50
*Y. lipolytica* Y11	18.75 ± 3.20	15.00 ± 2.00 *	19.00 ± 2.00	10.00 ± 0.00
*Y. lipolytica* Y12	13.75 ± 1.70	8.00 ± 2.00 *	10.75 ± 0.20	10.75 ± 0.20
*Y. lipolytica* Y13	14.25 ± 1.20	13.00 ± 1.00 *	12.75 ± 3.20	11.00 ± 2.00
S. boulardii *	21.15 ± 1.05	0.00 ± 0.00	18.25 ± 2.15	19. 05 ± 2.25

The values show diameters (mm) for inhibition zones. *: indicates statistically significant difference between control and all experimental groups as evaluated by Student’s *t*-test (*p* < 0.05). *S. boulardii*: control.

**Table 4:** Hemolytic activity and antibiotic susceptibility *of Y. lipolytica* strains obtained drom cold-pressed olive oil and *S. boulardii*.

Strains	Hemolytic Activity	Erythromycin (15 µg)	Gentamicin (120 μg)	Tetracycline (30 μg)	Vancomycin (30 μg)
*Y. lipolytica* Y1	γ	S	S	S	S
*Y. lipolytica* Y2	γ	S	S	S	S
*Y. lipolytica* Y3	γ	S	S	S	S
*Y. lipolytica* Y4	γ	S	S	I	S
*Y. lipolytica* Y5	γ	S	S	S	S
*Y. lipolytica* Y6	γ	S	S	S	S
*Y. lipolytica* Y7	γ	S	S	S	S
*Y. lipolytica* Y8	γ	S	S	S	S
*Y. lipolytica* Y9	γ	S	S	S	S
*Y. lipolytica* Y10	γ	S	I	S	S
*Y. lipolytica* Y11	γ	S	S	S	S
*Y. lipolytica* Y12	γ	S	S	S	S
*Y. lipolytica* Y13	γ	S	S	S	S
S. boulardii *	γ	S	S	S	S

*: control, γ-hemolysis: no zone, S: susceptible, and I: Intermate (National Committee for Clinical Laboratory Standards).

**Table 5:** Cholesterol removal (%) and DPPH radical scavenging activity (%) of *Y. lipolytica* strains obtained from cold-pressed olive oil and *S. boulardii*.

Strains	Cholesterol Removal (%)	DPPH Radical Scavenging Activity (%)	Trolox Equivalent (μM)
*Y. lipolytica* Y1	17.37 ± 0.03	12.60 ± 0.05	34.24 ± 0.13
*Y. lipolytica* Y2	37.78 ± 0.44	27.62 ± 0.97	75.07 ± 2.63
*Y. lipolytica* Y3	47.05 ± 0.26	39.10 ± 0.84	106.27 ± 2.28
*Y. lipolytica* Y4	39.69 ± 0.15	25.80 ± 0.65	70.12 ± 1.76
*Y. lipolytica* Y5	34.08 ± 0.18	7.43 ± 0.66	20.19 ± 1.79
*Y. lipolytica* Y6	47.42 ± 0.09	4.88 ± 0.76	13.26 ± 2.06
*Y. lipolytica* Y7	36.51 ± 0.16	6.51 ± 0.20	17.69 ± 0.54
*Y. lipolytica* Y8	40.33 ± 0.03	5.33 ± 0.44	14.48 ± 1.19
*Y. lipolytica* Y9	35.46 ± 0.10	13.42 ± 0.55	36.47 ± 1.49
*Y. lipolytica* Y10	12.30 ± 0.11	7.41 ± 0.34	20.14 ± 0.92
*Y. lipolytica* Y11	33.60 ± 0.62	2.85 ± 0.32	7.74 ± 0.86
*Y. lipolytica* Y12	29.08 ± 0.41	6.49 ± 0.14	17.64 ± 0.38
*Y. lipolytica* Y13	25.78 ± 0.28	5.31 ± 0.06	14.43 ± 0.16
S. boulardii *	86.74 ± 0.64	70.42 ± 0.54	191.41 ± 1.46

*: indicates statistically significant difference between control and all experimental groups as evaluated by Student’s *t*-test (*p* < 0.05). *S. boulardii*: control.

## Data Availability

The original contributions presented in the study are included in the article, further inquiries can be directed to the corresponding author.
